# Chromatic symphony of fleshy fruits: functions, biosynthesis and metabolic engineering of bioactive compounds

**DOI:** 10.1186/s43897-024-00142-y

**Published:** 2025-04-02

**Authors:** Huimin Hu, Nirakar Pradhan, Jianbo Xiao, Rui Xia, Pan Liao

**Affiliations:** 1 State Key Laboratory for Conservation and Utilization of Subtropical Agro-Bioresources, College of Horticulture, South China Agricultural University, Guangzhou, China; 2 Guangdong Laboratory for Lingnan Modern Agriculture, South China Agricultural University, Guangzhou, China; 3 Department of Biology, Hong Kong Baptist University, Kowloon Tong, Hong Kong SAR China; 4 Department of Analytical and Food Chemistry, Faculty of Sciences, Universidade de Vigo, Nutrition and Bromatology Group, Ourense, Spain; 5 State Key Laboratory of Environmental and Biological Analysis, Hong Kong Baptist University, Hong Kong SAR, China; 6 State Key Laboratory of Agrobiotechnology, The Chinese University of Hong Kong, Hong Kong SAR, China

**Keywords:** Fleshy fruits, Natural pigments, Human health, Biosynthetic pathways, Host cells, Metabolic engineering

## Abstract

Fleshy fruits are popular among consumers due to their significant nutritional value, which includes essential bioactive compounds such as pigments, vitamins, and minerals. Notably, plant-derived pigments are generally considered safe and reliable, helping to protect humans against various inflammatory diseases. Although the phytochemical diversity and their biological activities have been extensively reviewed and summarized, the status of bioactive nutrients in fleshy fruits, particularly with a focusing on different colors, has received less attention. Therefore, this review introduces five common types of fleshy fruits based on coloration and summarizes their major bioactive compounds. It also provides the latest advancements on the function, biosynthesis, and metabolic engineering of plant-derived pigments. In this review, we emphasize that promoting the consumption of a diverse array of colorful fruits can contribute to a balanced diet; however, optimal intake levels still require further clinical validation. This review may serve as a useful guide for decisions that enhance the understanding of natural pigments and accelerate their application in agriculture and medicine.

## Introduction

Fruits play a pivotal role in the dietary practices of humans due to their indispensable bioactive nutrients and regulatory functions. In general, angiosperms exhibit a wide variety of fruit types, including dry and fleshy fruits (Xiang et al. 2024). The development and ripening of fleshy fruits involve complex metabolic changes, primarily characterized by the accumulation of soluble sugars, pigments, and flavor compounds (Zhu et al. 2022). The types and concentrations of these metabolites not only reflect the physiological functions of fruit trees during growth and development, but also are closely relate to fruit quality, which directly influences the flavors and nutritional properties of fruits. Notably, the richness of color in fleshy fruits is significantly affected by the types and concentrations of biosynthesized pigmented natural products, as well as by co-pigments, metal ions, light conditions, and pH levels (Chandra et al. 2021).

The coloration of fruits is regarded as a crucial quality trait of fleshy fruits, as the pigments responsible for imparting color are closely associated with fruit flavor, health, and nutritional properties (Gebretsadik et al. 2021). Plant pigments can be classified into four categories: flavonoids, betalains, carotenoids, and chlorophylls. Among these, flavonoids are the most abundant phenolics in plants and exhibit multiple significant biological and pharmacological activities including anti-cancer, anti-allergic, anti-inflammatory, antioxidant, and anti-oedemic effects (Tao et al. 2022). Notably, anthocyanins are a type of flavonoids that exhibit the broadest range of colors, spanning from pale yellow to blue hues (Decker et al. 2024). Betalains are a group of natural red pigments that possess a unique nitrogenous core structure. They are found exclusively in families of the Caryophyllales order, as well as in some higher-order fungi, and have the ability to replace the otherwise ubiquitous anthocyanins in these organisms (Abedi-Firoozjah et al. 2023; Winkler et al. 2024). Carotenoids, the most abundant pigment produced by nature, are necessary compounds of photosystems and contribute to the yellow-to-red coloration observed in fruits (Sun et al. 2022; Zhang et al. 2024b). Chlorophylls are vivid chromophores that absorbs and use light in photosynthesis, and some chlorophyll derivatives display multiple health-promoting properties (Yang et al. 2024b). Recently, pigments from fleshy fruits have emerged as bioactive compounds given their potential health-promoting benefits, which have been extensively used in all aspects of daily life (Maheshwari et al. 2022; Tomas et al. 2022).

Various factors, including appearance (particularly colors and textures), flavor, and nutritional profiles, influence consumer’s decisions when selecting fruits (Cao et al. 2024; Wang et al. 2022a). Hence, a common question from consumers is whether fruits with similar colors also have similar nutritional value. Answering this question is valuable for helping consumers to make informed choices in fruit selection and receive scientifically sound dietary recommendations. Therefore, it is necessary to compile the metabolic differences and nutrient contents of different colorful fleshy fruits. Although phytochemical diversity and biological activities have been extensively reviewed and summarized, the status of bioactive nutrients in fleshy fruits focusing on different colors is less reviewed. Therefore, the objectives of the review are to elucidate the importance of exploring the diverse benefits offered by pigmented fleshy fruits, provide recommendations for the human diet, update the latest progress on the function, biosynthesis, and metabolic engineering of these bioactive compounds, and propose future perspectives in this area based on the current challenges faced in utilizing pigmented natural products.

## Color diversity of the fleshy fruits

Colorful fleshy fruits are abundant sources of natural bioactive compounds. Different colors of fleshy fruits have different health-promoting effects due to various types and contents of bioactive compounds (Table 1). According to different colors, fleshy fruits can be categorized into five main groups: red, orange/yellow, green, white, and blue-black.

**Table 1:** The contents of major antioxidant phytochemicals of selected fruits

Flesh color	Species	Total phenolic content	Total flavonoid content	Total anthocyanidin content	Antioxidant activity	References
Red	Cherry tomato	1850.2^a^	1691.8^a^	High in purple cherry tomato	High	(Bhandari et al. 2016)
	Strawberry	200–400^b^	100–200^c^	0.17–0.22^d^	High	(ParraPalma et al. 2020; Wolfe et al. 2008)
	Red dragon fruit	390^e^	-	-	High	(Chen et al. 2021)
	Blood orange	High	682.95^f^	101.06^g^	High	(FornerGiner et al. 2023)
	Watermelon	390–740^h^	270–360^i^	15.93–54.16^j^	High	(Bazié et al. 2022)
Orange/yellow	Lemon	349^k^	146^l^	-	High	(Dong et al. 2019)
	Mandarin	12.79- 48.71^m^	2.08–7.57^n^	-	High	(Singh et al. 2021)
	Yellow-fleshed kiwifruit	674^o^	15–50^p^	-	High	(Li et al. 2018; Yuan et al. 2023)
Green	Green-fleshed Kiwifruit	529^q^	40–80^r^	-	High	(Li et al. 2018; Yuan et al. 2023)
	Avocado	410.2^s^	21.9^t^	-	High	(Vinha et al. 2013; Wolfe et al. 2008)
White	Lychee	1.84^u^	0.49^u^	0.75^u^	High	(Xie et al. 2022)
	White strawberry	145.8–262.1^v^	-	1.59–7.86^w^	High	(Noriega et al. 2021)
Blue-black	Blueberry	319.3^x^	-	131.2^x^	High	(Wang et al. 2008; Wolfe et al. 2008)
	Blackcurrant	26.2^y^	24.43^z^	3.68^aa^	High	(Blejan et al. 2023; Wolfe et al. 2008)

'-' indicates that it was not mentioned in the research

^a^Mean values were expressed in mg kg^−1^ on a dry weight basis of commercial cultivars

^b^Mean values were expressed in mg GAE/100 g on a fresh weight basis of three different strawberry cultivars from western Portugal region. Quantification was performed based on a standard curve of gallic acid. GAE, gallic acid equivalent

^c^Mean values were expressed in mg QE/100 g on a fresh weight basis of three different strawberry cultivars from western Portugal region

^d^Mean values were expressed in g/kg Cy3G on a fresh weight basis of three different strawberry cultivars from western Portugal region. Cy3G, cyanidin 3-glucoside equivalent

^e^Mean values were expressed in mg GAE/100 g of red dragon fruit pulp. Quantification was performed based on a standard curve of gallic acid

^f^Mean values were expressed in mg/L on the juice of blood oranges varieties ‘Moro’ (*Citrus sinensis* (L.) cv. Moro)

^g^Mean values were expressed in mg/L on the juice of blood oranges varieties ‘Sanguinelli’ (*Citrus sinensis* (L.) cv. Sanguinelli)

^h^Mean values were expressed as mg gallic acid equivalent (mg GAE) per 100 g of dry matter among five watermelon cultivars

^i^Mean values were expressed as mg quercetin equivalent (mg QE) per 100 g of dry matter among five watermelon cultivars

^j^Mean values were expressed as mg CE/g. CE, cyanidin-3-glucoside equivalent

^k^Mean values were expressed in mg GAE/100 g on a fresh weight basis of Eureka lemon fruits (*Citrus limon* (L.) Burm. f.)

^l^Mean values were expressed in mg RE/100 g on a fresh weight basis of Eureka lemon fruits (*Citrus limon* (L.) Burm. f.). RE, rutin equivalent

^m^Mean values were expressed in mg GAE/100 g on a fresh weight basis on pulp of Kinnow and W. Murcott mandarins

^n^Mean values were expressed in mg RE/100 g on a fresh weight basis on pulp of Kinnow and W. Murcott mandarins

^o^Total phenolic content was expressed as mg gallic acid equivalent (mg GAE) per 100 g of dry matter of Xuxiang (*A.chinensis* var. *deliciosa*)

^p^Total flavonoid content was expressed as mg quercetin equivalent (mg QE) per 100 g of dry matter among six yellow-fleshed kiwifruit cultivars

^q^Total phenolic content was expressed as mg gallic acid equivalent (mg GAE) per 100 g of dry matter of Jinyuan (*A.eriantha* × *A.chinensis*)

^r^Total flavonoid content was expressed as mg quercetin equivalent (mg QE) per 100 g of dry matter among six green-fleshed kiwifruit cultivars

^s^Mean values were expressed in mg GAE/100 g on a fresh weight basis of avocado fruit

^t^The flavonoid content was expressed in milligrams per 100 g of fresh weight

^u^Mean values were expressed in mg/g of litchi (*L. chinensis* Sonn.) of the “Feizixiao” variety

^v^Mean values were expressed as mg of gallic acid equivalents per 100 g of fresh fruits (mg GAE/100 g FW)

^w^Mean values were expressed as mg equivalents per 100 g of fresh fruits (mg Eq 100 g^−1^ FW)

^x^Mean values were expressed in mg/100 g on a fresh weight basis of organically cultured fruit

^y^Mean values were expressed as mg GAE per 100 g dry pomace sample

^z^Mean values were expressed as mg of QE per gram of dry pomace

^aa^Mean values were expressed as milligrams of cyanidin-3-glucoside equivalents (CGE) per gram of dry pomace

### Major nutrients and bioactive compounds in red fruits

**Fig. 1:**
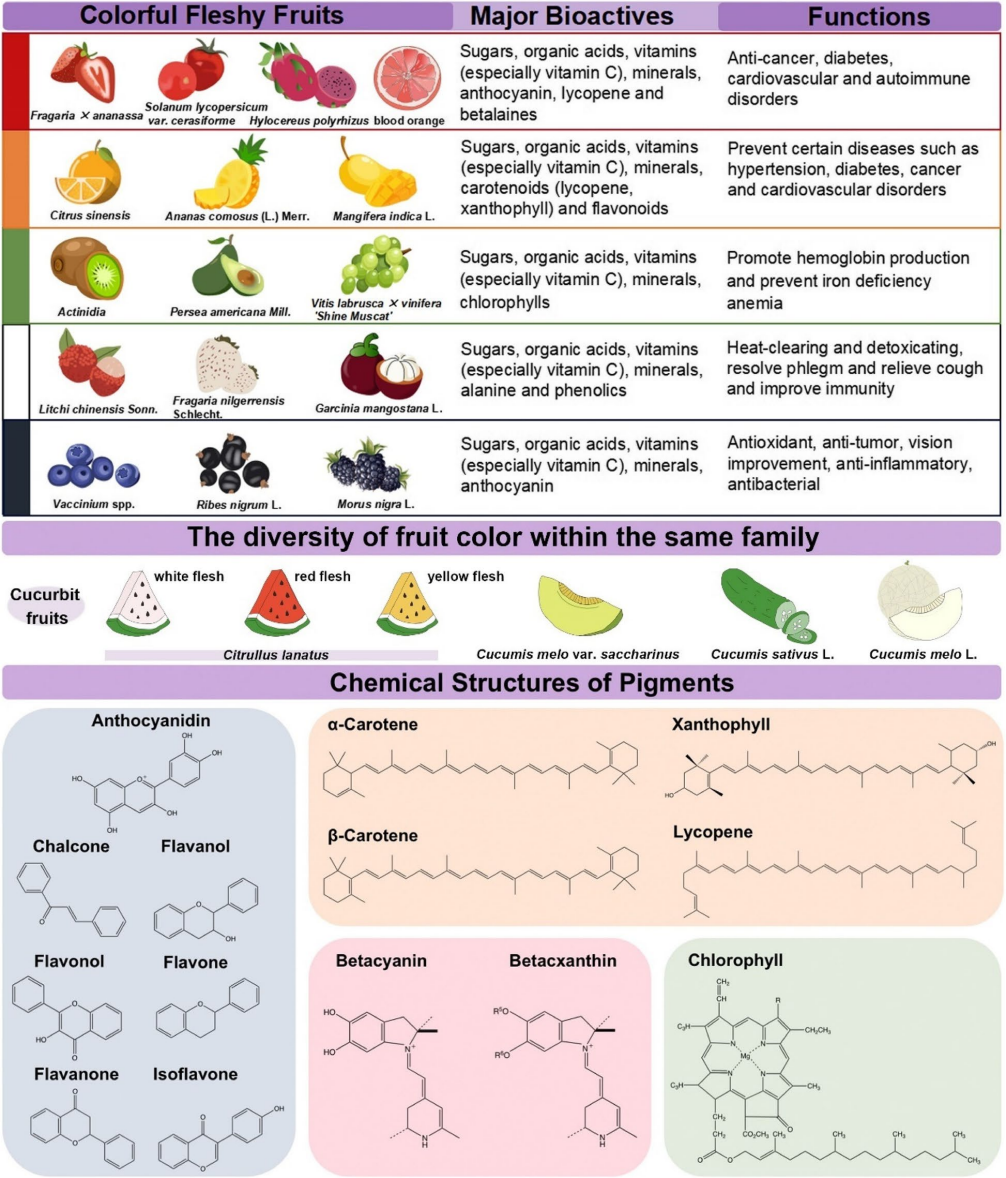
Chemical structures of major pigments in fleshy fruits. Created with Adobe Illustrator and Chemdraw 20.0

Cherry tomato (*Solanum lycopersicum* var. *cerasiforme*), strawberry (*Fragaria* × *ananassa*), red dragon fruit (*Hylocereus polyrhizus*, red pulp with pink peel), and blood orange (*Citrus sinensis*) are regarded as representatives of the red fruit kingdom (Fig. 1). Cherry tomato is one of the economically important fruits in the world, which belongs to the tomato genus *Solanum* (Guo et al. 2021). In mature regular tomatoes, about 75% of the dry matter is derived from solids including sugars, organic acids, minerals, and pectin which account for ∼50%, > 10%, ∼7%, and 8% respectively (Wu et al. 2022a). Compared with regular tomatoes, cherry tomatoes are richer in vitamins, which is 1.7 times than the regular tomatoes. Likewise, lycopene and other bioactive nutrients in cherry tomatoes are more abundant than the regular tomatoes (Wang et al. 2022b; Zeng et al. 2020). Strawberries from both subtropical and temperate zones are known to contain a variety of valuable bioactive compounds, such as sugars, vitamin C, minerals and polyphenols (Newerli-Guz et al. 2023). Anthocyanin and lycopene are the major pigments from different categories that were determined in strawberry fruits (Šic Žlabur et al. 2020). It is reported that the total anthocyanin content ranged between 200 and 600 mg kg^−1^ in strawberry fruits from five different cultivars (da Silva et al. 2007). Red dragon fruit, originally from tropical and subtropical zones, is a highly popular fruit with certain health benefits, for instance as its anticancer and antinociceptive properties (Marques et al. 2023). Several compounds contribute to the pigmentation in red dragon fruit, such as flavonoids and betalaines, which contribute to its antioxidant activity. Among these, the levels of flavonoids and betacyanins presented as betanin equivalents per 100 g fresh flesh were 10.3 ± 0.22 mg and 7.21 ± 0.02 mg, respectively (Wu et al. 2006). Blood orange is a rare red-fleshed sweet orange with a high anthocyanin content and serves as an excellent source of natural antioxidants and bioactive compounds, including flavonoids, anthocyanins, phenols and ascorbic acid. Previous study analyzed the content of anthocyanins of the eleven blood orange juice and found that the highest level of total anthocyanins was in *Citrus sinensis* (L.) cv. Moro (133.10 mg L^−1^) (Habibi et al. 2023; Legua et al. 2022).

### Major nutrients and bioactive compounds in orange/yellow fruits

As for the color of orange or yellow, there are many fruits that come into sight, especially citrus (genus *Citrus* L.) fruits (Fig. 1), such as orange (*Citrus sinensis*), lemon (*C. limon*), mandarin (*C. reticulata*), etc. They are rich in various bioactive products such as carotenoids, flavonoids, limonoids, terpenoids, and synephrines (Lu et al. 2023). *Citrus* carotenoids and flavonoids possess biological effects against cancer and cardiovascular diseases (Allister et al. 2008; Yi et al. 2017). Notably, the natural limonoids exhibit a broad range of biological activities such as antioxidant and anti-inflammatory (De La Peña et al. 2023). Synephrine, a major phytochemical found in bitter orange, is widely used as a dietary supplement for weight loss and fat reduction (Dodonova et al. 2023). Moreover, citrus fruits are recognized as a significant dietary source of vitamin C for humans (Lu et al. 2023). A previous study indicated that the presence of carotenoids confers the orange-yellow or orange-red color to the pulp and peel of citrus fruits (Luan et al. 2020). Carotenoids are the major class of bioactive compounds present in citrus fruits, of which the most abundant carotenoid is xanthophyll, particularly β,β-xanthophylls (Zacarías-García et al. 2021). It is reported that around 115 carotenoids in citrus species, which can be divided into two categories: (i) carotenes that are nonoxygenated tetraterpenoids such as α-carotene, β-carotene, and lycopene, and (ii) oxygen-containing xanthophylls including zeaxanthin, β-cryptoxanthin, and violaxanthin (Ikoma et al. 2016). In citrus fruits, β-cryptoxanthin is a precursor of vitamin A, which is beneficial to the health of human beings (Burri 2015). Furthermore, citrus fruits exhibit significant variation in the types and concentrations of carotenoids based on their cultivars, maturity stage, environmental factors, and growth conditions (Ledesma-Escobar et al. 2018; Peng et al. 2021).

### Major nutrients and bioactive compounds in green fruits

Kiwifruit (*Actinidia*), avocado (*Persea americana* Mill.), and green grape (e.g., *Vitis labrusca* × *vinifera 'Shine Muscat'*) are green fleshy fruits (Fig. 1), which have economic and medicinal value because they contain a variety of bioactive compounds. A study on the green-fleshed *Actinidia deliciosa* found that the soluble solids content ranged from 12 to 20%, with an average of 16%. Additionally, the vitamin C content ranged from 54.86 to 159.08 mg/100 g fresh weight (FW), with an average of 87.19 mg/100 g FW (Ma et al. 2017). Recently, various kiwifruit varieties with different flesh colors, such as red and yellow, have gained popularity among consumers. The variation in flesh color primarily depends on the types and concentrations of pigments present. Red and yellow varieties contain higher levels of carotenoids during the whole fruit development period, while they show a higher chlorophylls/carotenoids ratio (≥ 6.0) during the late developmental stage contributes to the formation of green color in kiwifruit flesh, indicating that the green fleshy kiwifruit contains more chlorophylls. Avocado is a tropical fruit with high nutritional value, rich in various vitamins, fatty acids, proteins, and minerals, and has good health effects. Recent work has measured the nutrient and phytochemical composition of the ripe fruit of avocado, quantifying seven organic acids, six sugars, nine fatty acids, eleven carotenoids, three phytosterols, one phytostanol, five chlorophylls, and related compounds, two tocopherols, and several phenolic compounds in the tested pulps (Ramos-Aguilar et al. 2021). These nutrients play an important role in anti-oxidation, preventing diabetes and cardiovascular diseases. Among these compounds, chlorophylls and their derivatives have strong antioxidant and anticancer functions (Åhlberg 2022; Ramos-Aguilar et al. 2021).

### Major nutrients and bioactive compounds in white fruits

Lychee (*Litchi chinensis* Sonn.), white strawberry (*Fragaria nilgerrensis* Schlecht.), and mangosteen (*Garcinia mangostana* L.) have different appearances and colors. Nevertheless, they all possess a white pulp and taste sweet (Fig. 1). Recent result showed that lychee is beneficial to human health because it contains a variety of vitamins, phenolics, polysaccharides, and minerals (Hussain et al. 2021). For instance, various studies have reported that the main nutritional component in lychee pulp is sugar, such as glucose, sucrose, and fructose, while the levels of sucrose are roughly equal to that of fructose (Zhao et al. 2020). The content of sugars is between 10 and 19.2%, while reducing sugars account for 70% (Hajare et al. 2013; Wang et al. 2022c; Zhao et al. 2017). Subsequently, Tan et al. (2022) identified and quantified nine phenolic compounds using UPLC-QQQ-MS/MS, and naringin, rutin as well as p-coumaric acid were found to be the main phenolic products in all the lychee cultivars tested. White strawberry is a popular strawberry that produces white fruits with distinctive aromas, and its pulp contains vitamin C, sugars, protein, amino acids, dietary fiber, carbohydrates and other nutrients. A study reported that among different varieties, white strawberries had the highest fructose and glucose content, 4.73% and 4.30%, respectively. Moreover, white strawberries have high phenolics content and thereby show strong antioxidant activity (Noriega et al. 2021). Indeed, the strong antioxidant capacity of white strawberries arise from the accumulation of non-pigment antioxidants (especially vitamin C) and environmental factors (Li et al. 2019b). These factors compensate for the lack of pigments, allowing white strawberries to exhibit substantial antioxidant activity under certain conditions. The mangosteen pulp is rich in health-promoting nutrients and phytochemicals such as prenylated and oxygenated xanthones, flavonols, flavonoids, anthocyanins, tannins, carotenoids, ascorbic acid, and other bioactive natural products (Palakawong and Delaquis 2018). It has been reported that the protein, fatty acid, and carbohydrate content in dried mangosteen fruit is 0.6%, 1.9%, and 6.5%, respectively. Additionally, mangosteen fruits are particularly high in the content of Ca, K, Mg, alanine, and glutamic acid (Li et al. 2021).

### Major nutrients and bioactive compounds in blue-black fruits

Fruits with mysterious blue-black flesh include blueberries (*Vaccinium* spp.), blackcurrants (*Ribes nigrum* L.) and mulberries, which have attracted the attention of scholars because of their abundant bioactive products (Fig. 1). Blueberry is regarded as a ‘superfood’ due to its richness in anthocyanins, flavonoids, polysaccharides, vitamins, minerals and other nutrients in the flesh. In comparison to other fruits, blueberries contain a higher phenolic content, especially anthocyanins (Li et al. 2021; Liu et al. 2024; Sivapragasam et al. 2023). Initially, a study found that the total phenol amount of blueberry fruit in North America was 393 mg/100 g of edible portion, and the content of vitamin C is 9.7 mg/100 g of edible portion (Mazza 2005). Subsequently, Jeong et al. (2008) confirmed that the total phenol level of blueberry fruit in Korea was 902 mg/100 g fresh weight. Additionally, blackberries have a strong antioxidant capacity due to their high levels of anthocyanins in the pulp (Higbee et al. 2022). Recently, some research discovered that mulberry contains not only a lot of vitamins, free acids, and minerals but also anthocyanins, polysaccharides, resveratrol, and other bioactive components. Hao et al. (2022) reported that among the biologically active constituents, phenolic compounds, specifically flavonoids, anthocyanins, and phenolic acids, were found to be noteworthy. Building upon this, similar findings proposed that the α-glucosidase inhibitory effect and antioxidant activity of the mulberry fruit were correlated positively with the total amount of formed phenolic compounds (Dou et al. 2022). Therefore, blueberry, blackcurrant, and mulberry are functional fruits with high edible and health-promoting values due to their abundant bioactive phenolic compounds.

### The diversity of fruit color within the same family

The Cucurbitaceae family includes many economically important fruit crops, which are highly consumed for their diverse colors and abundant nutritional value (Davidson et al. 2023; Ma et al. 2022). These fruits display a wide range of colors, such as red (e.g., certain watermelon), orange/ yellow (e.g., Hami melon), green (e.g., cucumber), and white (e.g., honey dew melon) (Fig. 1). Cucurbit fruits are packed with essential nutrients, and are also rich in carotenoids, phenolic compounds, terpenoids, and phytochemicals (Nyirahabimana et al. 2022). Among these bioactive compounds, carotenoids can help reduce oxidative stress and may lower the risk of chronic diseases like cardiovascular disease and cancer (Ma et al. 2022). Previous study found that carotenoid contents (prolycopene, lycopene, β-carotene, ζ-carotene, and neoxanthin) determined different fruit flesh colors among 14 watermelon cultivars (Jin et al. 2019). Importantly, cucurbitacin B, a natural compound extracted from *Cucumis melo* var. *Cantalupensis* controls lung cancer cell proliferation and apoptosis (Liu et al. 2022). Collectively, regular consumption of cucurbit fruits has been associated with anti-inflammatory effects and improved metabolic health, making them a valuable addition to a balanced diet.

## The function of fruit pigments

As shown in Fig. 1, according to their chemical structures, flavonoids, carotenoids, betalaines, and chlorophylls are known as the four major pigments in the fleshy fruit kingdom, which cause a variety of fruit colors. In the visible light region, these pigments have a specific reflection spectrum, and the wavelength determines the color of the reflected light. For instance, green pigments such as chlorophyll reflect the green part of the spectrum but also absorb longer wavelengths of red and yellow, and shorter wavelengths of blue.

### Flavonoids

Flavonoids, a class of secondary metabolites, are phenolic compounds that exhibit a wide range of colors, varying from pale yellow to blue (Tanaka et al. 2008). In terms of chemical composition, flavonoids have a fundamental framework consisting of three interlinked rings (C6-C3-C6) (Geissman and Hinreiner 1952; Xu et al. 2024). These compounds can be categorized into seven primary subclasses, namely anthocyanidins, chalcones, flavanols, flavonols, flavanones, flavanols, and isoflavones, based on their distinctive structural variations (Shen et al. 2022b; Veitch and Grayer 2008) (Fig. 1). Among of these compounds, anthocyanidins are the most widely distributed flavonoids in the plant kingdom, commonly used as natural food colorants and can confer red, purple, and blue colors to fruits.

Anthocyanins are rich in nutrients and possess extensive medicinal value, such as antioxidant, anticancer, improve visual acuity, modulate the intestinal microbiota, antibacterial and anti-inflammatory, and other beneficial biological activities (Yousuf et al. 2016). Previous research reported the impact of blackcurrant anthocyanins on endothelial function in young smokers revealed that the administration of a supplement containing 50 mg of blackcurrant anthocyanins alongside vitamin E exhibited the potential to alleviate the acute endothelial dysfunction caused by smoking (Tomisawa et al. 2019). In another study, it was discovered that 12-week supplementation with 80 mg/day or more of anthocyanins can reduce platelet function in individuals with dyslipidemia to reduce the morbidity of thrombosis (Tian et al. 2021). Recently, it was evaluated that the efficacy of anthocyanins in mitigating the risk of chronic diseases resulting from glycation and inflammation (Gao et al. 2023). The research mentioned above found that these bioactive compounds demonstrated considerable anti-glycation and anti-inflammatory activities. Hence, anthocyanins hold significant potential as health-promoting phytochemicals that can effectively mitigate the development of such ailments. Their impact on cardiovascular health and lung function has been extensively documented in various research studies (Cassidy et al. 2016; Liu et al. 2023b). Although we have known the beneficial effects of anthocyanins, a large number of animal and clinical studies are still in progress to explore its best curative mechanism.

On one hand, anthocyanins can be used as functional dietary antioxidants and are beneficial to human health (Choudhary et al. 2023). On the other hand, they can be used as natural colorants in the food, cosmetics, and the pharmaceutical industries. A study proposed that a health-enhancing food with abundant anthocyanins could improve vascular health (Fairlie-Jones et al. 2017). Furthermore, the pH-responsive color-changing properties of anthocyanins make them extremely valuable in the development of intelligent and active food packaging with color indicator capabilities (Roy and Rhim 2021). This distinctive feature enables anthocyanins to undergo color transformation in response to variations in the pH level of the food or its surrounding environment, thereby offering visual cues for assessing freshness, spoilage, and other quality parameters. Thus, by integrating anthocyanins into food packaging materials, manufacturers can create innovative packaging solutions that effectively enhance food safety, extend shelf life, and improve the overall consumer experience (Roy and Rhim 2021; Wang et al. 2023b).

### Carotenoids

Carotenoids are a kind of natural tetraterpene pigments, which are responsible for colors ranging from yellow through orange to red due to the system of conjugated double bonds (Cesarino 2022). Typically, carotenoids have a 40-carbon chain backbone composed of eight isoprene molecules (Britton 1995). There are over 1,100 known carotenoids belonging to two main groups, xanthophylls and carotenes, which differ only in their oxygen content (Yan et al. 2024). Meanwhile, other kinds of carotenoids are found in daily diets, such as lycopene, β-cryptoxanthin, and zeaxanthin.

Xanthophyll is a bioactive carotenoid known as hydrocarbons and found in orange and yellow fruits, such as orange, lemon, mango. Xanthophyll possesses antioxidant, anti-inflammatory, and immunity-boosting properties and other benefits, among which the protection of the retina is the most prominent (Sharma et al. 2024). For instance, a research succinctly outlined the selective accumulation of zeaxanthin, lutein, and xanthophylls within the macula, providing protection against age-related macular degeneration (Thomas and Harrison 2016). Subsequently, it is reported that xanthophylls effectively safeguard visual functions against the deleterious effects of blue light, singlet oxygen, and triplet photosensitizers (Skibsted 2018). Nevertheless, there exists a measure of apprehension surrounding the possibility that higher dosages of xanthophylls may be associated with an augmented susceptibility to skin cancer and gastric adenocarcinoma (Aziz et al. 2020).

Another major carotenoid is carotene, which is lipid-soluble and insoluble in water and can be made up of α, β, γ, δ, ε, and ζ-carotene. Lycopene, a type of carotene, generally exists in red fruit. The antioxidant activities of lycopene and its scavenging effect on lipid peroxides, nitric oxide, and reactive oxygen species are due to it containing eleven conjugated double bonds (Tripathi et al. 2024; Breemen and Pajkovic 2008). A growing body of research has consistently highlighted the potential benefits of lycopene in mitigating chronic diseases, such as certain types of cancers and coronary heart disease (Li et al. 2024). Previous investigation identified that lycopene effectively suppressed lipid accumulation in palmitate-treated HepG2 hepatocytes cell lines by upregulating PPARα expression and ameliorating mitochondrial function (Wang et al. 2020). Furthermore, a randomized clinical trial conducted demonstrated that lycopene supplementation enhanced endothelial function and reduced triglyceride levels in patients with ischemic heart failure with reduced ejection fraction (HFrEF) (Karimian et al. 2022).

Carotenoids, especially lycopene, are emerging as a kind of valued antioxidant, with multiple applications as a nutritional supplement and an active ingredient in cosmetic products (Guerra et al. 2021). Meanwhile, as a natural coloring compound, it can replace food colorants to avoid harmful effects (Prihastyanti et al. 2021). Notably, carotenoids undergo enzyme-catalyzed oxidative cleavage reactions and non-enzymatic degradation processes, resulting in the production of different carbonyl products referred to as apocarotenoids (Hou et al. 2016; Sun et al. 2022). Some apocarotenoid pigments, such as crocins, picrocrocin and bixin, are extensively utilized as colorants and additives in the food and pharmaceutical industries (Zheng et al. 2021). Additionally, it is reported that the engineered tomatoes (engineering high levels of saffron apocarotenoids in tomato) exhibited higher antioxidant capacity and were able to protect against neurological disorders (Ahrazem et al. 2022).

### Betalaines

Betacyanins are responsible for the red pigmentation in fruits, such as red dragon fruit (Tarte et al. 2023). Based on their chemical structure and light-absorption properties, betalains, which consist of the subgroups yellow betaxanthins and red-violet betacyanins. It has color effect on plants, helps plants adapt to adversity, and also plays an important role in osmotic adjustment. In addition, betaine can not only be used as a colorant for food and medicine, but also has medicinal effects due to its great antioxidant capacity. A comparative study on the intestinal effects of red beetroot consumption reported that betacyanins correlate positively with *Bifidobacterium* and *Coprococcus*, and inversely with *Ruminococcus*, this result emphasized the potential benefit of betacyanins on intestinal health (Wang et al. 2023a). Transgenic tomato fruits overexpressing betacyanin biosynthesis gene, *CYP76AD1*, showed better anti-inflammatory activity than wild-type fruits (Saito et al. 2023). Additionally, betacyanins proved to be a strong inhibitor in the growth of tumor cell lines, such lung, stomach, breast, colon, and other tumor cells. Betacyanins show a strong effect on inhibiting the growth of tumor cell lines, such as lung, stomach, breast, colon, and other tumor cells (Fernández-López et al. 2020). Owing to special pigment characteristics, betacyanins can be used as yogurt colorant (CaldasCueva et al. 2016) and intelligent packaging (Naghdi et al. 2021). Although some progress has been made in the medical value of betacyanins, no broad and consistent conclusions have been reached, so further research is needed.

### Chlorophylls

Chlorophyll is the natural compound that accounts for the green color present in the colorful fruit kingdom. Chlorophyll can be of two major types: chlorophyll a and chlorophyll b (Tanaka and Tanaka 2006). According to their chemical structures, the only difference between the two groups of chlorophyll is that chlorophyll a contains a methyl group, whereas chlorophyll b contains a formyl group. Apart from the above, chlorophyll c, d, e also exists in the plant kingdom. The most widely distributed form in terrestrial plants is chlorophyll a, followed by chlorophyll b, while chlorophyll c, d, and e are found exclusively in certain microalgae, algae and several photosynthetic bacteria.

Chlorophyll is not only essential to plant life, but also beneficial to human health. The major functions of chlorophyll include cleaning the intestines, healing the skin, preventing cancer, aiding weight loss, and stimulating the immune system. Previous research has elucidated that the inherent presence of chlorophyll in green vegetables could effectively function as a shield, safeguarding against the adverse, cytotoxic, and hyperproliferative repercussions on the colon posed by dietary haem (de Vogel et al. 2005). Moreover, a study undertook the eco-friendly synthesis of chlorophyll-functionalized carbon quantum dots (Chl-CQDs) via a hydrothermal process utilizing banana leaves. Their findings suggested these Chl-CQDs signified considerable therapeutic potential against neoplastic cells, whilst seemingly demonstrating no cytotoxicity towards normal cells (Alam et al. 2022). Additionally, due to their strong fluorescence signals coupled with low toxicity, chlorophyll derivatives serve as effective fluorescent tags for biological imaging (Li et al. 2017). On one hand, chlorophyll serves as an intrinsic natural chromophore, while also being incorporated into chewing gum for its deodorizing attributes. Derivatives of chlorophyll find widespread application as pharmaceutical constituents, effectively safeguarding human well-being.

The utilization of natural pigments in the food industry presents various challenges. These challenges include issues related to stability, production and extraction costs, sourcing and sustainability, food safety and regulatory compliance, consistency and stability in different food systems, sensory characteristics, and consumer acceptance of pigmented foods. To promote the widespread application of natural pigments in the food industry, further research and technological advancements are needed to address these challenges effectively.

## Biosynthesis

### Biosynthesis of anthocyanins

**Fig. 2:**
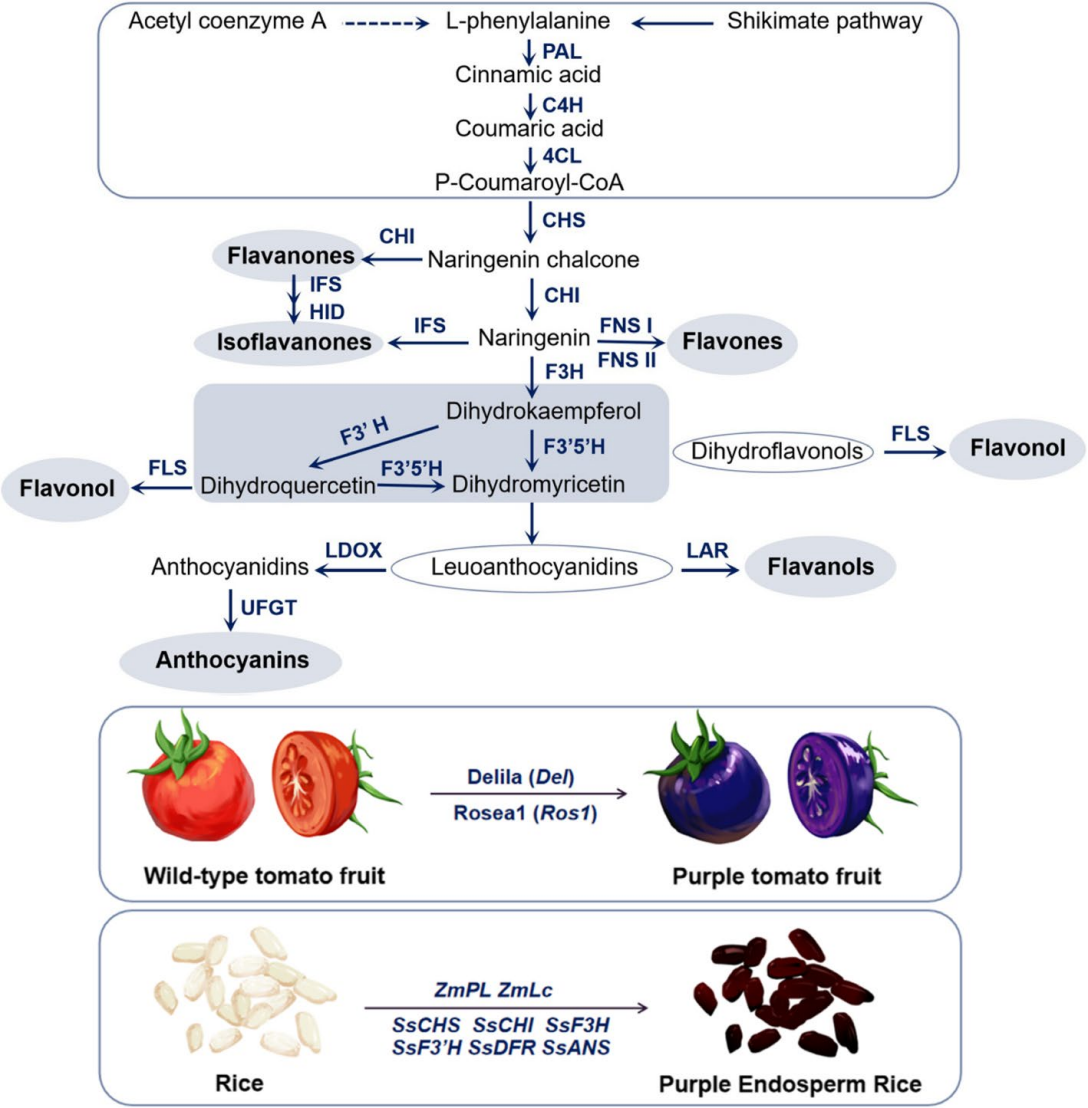
Flavonoid biosynthetic pathways. PAL, phenylalanine ammonia-lyase; C4H, cinnamic acid 4-hydroxylase; 4CL, 4-coumarate CoA ligase; CHS, chalcone synthase; CHI, chalcone isomerase; IFS, isoflavone synthase; HID, 2-hydroxyisoflavanone dehydratase; FNS, flavone synthase; F3H, flavanone 3-hydroxylase; F3′H, flavanoid 3′-hydroxylase; F3′5′H, flavanone 3′,5′-hydroxylase; FLS, flavonol synthase; LDOX, leucoanthocyanidin dioxygenase; LAR, leucoanthocyanidin reductase; UFGT, UDP-glucose flavonoid 3-O-glucosyltransferase

Flavonoids are the largest group of polyphenols. For plants, we summarize the flavonoid biosynthetic pathway in Fig. 2. The biosynthesis of flavonoid compounds is a complex process, with its metabolic pathways being governed by a chain of pivotal enzymes such as chalcone synthase (CHS), chalcone isomerase (CHI), and isoflavone synthase (FLS) (Holton and Cornish 1995; Shen et al. 2022b). The expression of essential enzyme genes within metabolic pathways exhibits a strong correlation with the levels of their corresponding synthesized metabolites. Using tomatoes as a model, the introduction of three foreign genes (Delila and Rosea1 genes from the *Antirrhinum majus*, and MYB12 gene from *Arabidopsis*) into wild-type tomatoes can effectively activate flavonol biosynthesis, resulting in the purple pigmentation of tomato fruits due to increased anthocyanin content (Butelli et al. 2008). Purple tomatoes have a longer shelf life and offer more health benefits compared to regular tomatoes. Rice is one of the most important food crops. In addition to increasing rice yield and developing new varieties that can combat harsh environments, scientists are particularly keen on making rice more nutritious. A research team designed genes associated with anthocyanin biosynthesis in rice endosperm and employed an efficient TransGene Stacking II (TGS II) vector toolkit for plant multigene stacking. They successfully produced a transgenic rice variety named ‘Purple Endosperm Rice’, which accumulates abundant anthocyanins in the endosperm (Zhu et al. 2017).

### Biosynthesis of carotenoids

**Fig. 3:**
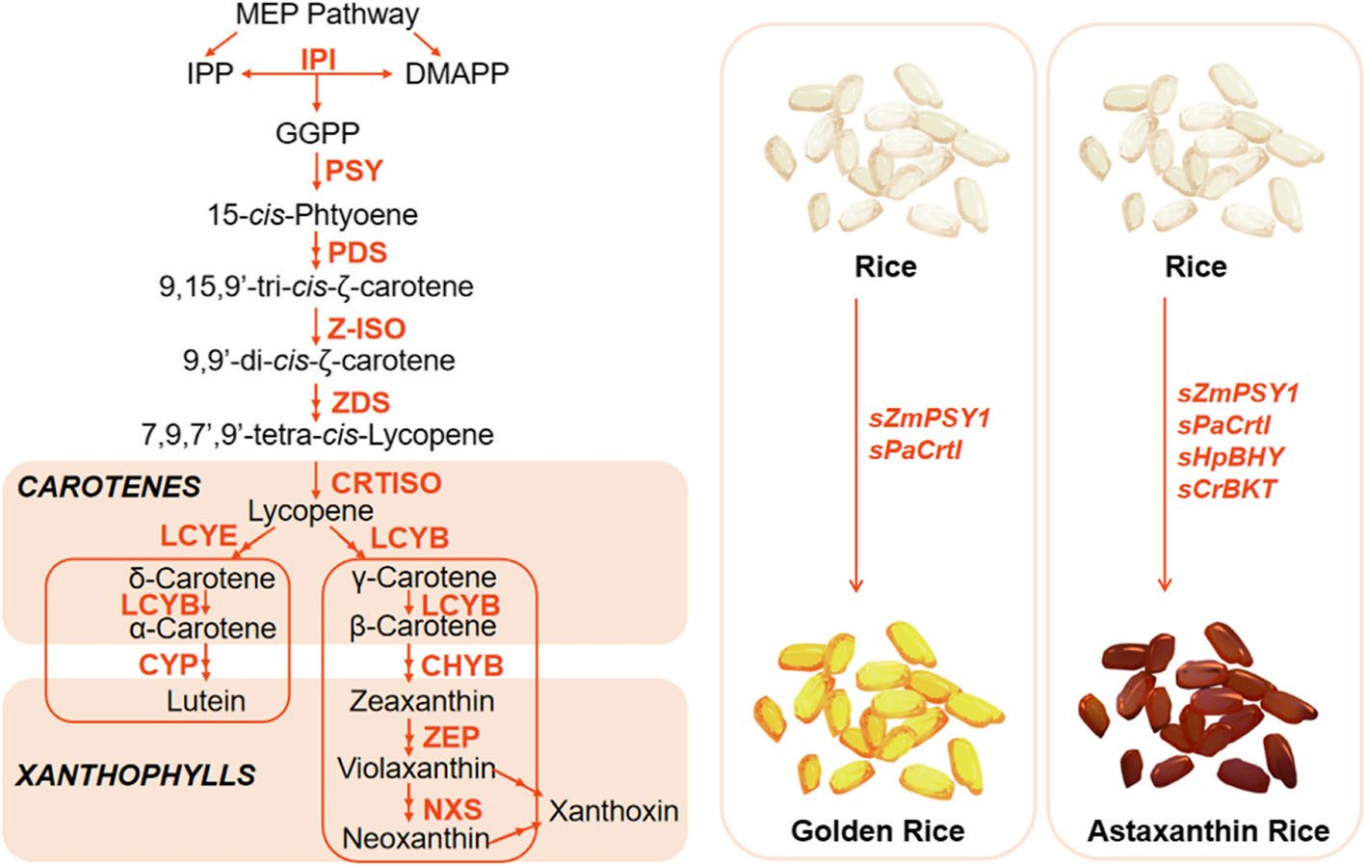
Carotenoid biosynthetic pathways. MEP, plastidial 2-*C*-methy1-D-erythrito1-4-phosphate pathway; IPP, isopentenyl diphosphate; DMAPP, dimethylallyl diphosphate; GGPP, geranylgeranyl diphosphate; IPI, isopentenyl diphosphate isomerase; GGPPS, GGPP synthase; PSY, phytoene synthase; PDS, phytoene desaturase; Z-ISO, ζ-carotene isomerase; ZDS, ζ-carotene desaturase; CRTISO, carotenoid isomerase; LCYE, lycopene ε-cyclase; LCYB, lycopene β-cyclase; CYP, Cytochrome P450 enzymes; CHYB, β-carotene hydroxylase; ZEP, zeaxanthin epoxidase; NXS, neoxanthin synthase; sZmPSY1, Zea mays PSY1; sPaCrtI, Pantoea ananatis (formerly named Erwinia uredovora) CrtI; sCrBKT, Chlamydomonas reinhardtii BKT; sHpBHY, Haematococcus pluvialis BHY. Multi-arrows represent multi-step reactions. The carotenoids are segregated into distinct groups by rectangles

As shown in Fig. 3, there are two biosynthetic pathways of carotenoids in plants: the cytosolic mevalonate (MVA) and plastidial 2-*C*-methy1-D-erythrito1-4-phosphate (MEP) pathways, both of which produce the precursors for the biosynthesis of carotenoids, C5 isopentenyl diphosphate (IPP) and dimethylallyl diphosphate (DMAPP) (Zhu et al. 2022). Also, GGPP generates the first carotenoid substance, 15-*cis*-phytoene, which is then transformed into other carotenoids through dehydrogenation, cyclization, hydroxylation, and epoxidation (Lu and Li 2008). This serves as a critical cornerstone upon which the diversity of carotenogenesis is built, manifesting itself in the vibrant spectrum of pigments integral to plant biology and nutrition science (Yonekura-Sakakibara et al. 2019). A previous study demonstrated the significant enhancement of β-carotene content in a novel genetically modified Golden rice developed by Syngenta (Paine et al. 2005). The Golden rice variant exhibits a β-carotene content approximately 23 times higher (maximum 37 μg/g) than that of regular rice and has been primarily engineered to combat vitamin A deficiency. Astaxanthin, a high-level carotenoid product, possessing vibrant orange color and potent free radical scavenging capabilities, manifests anti-aging and cardiovascular disease prevention properties. The research team devised a de novo astaxanthin biosynthesis pathway within the endosperm of rice and successfully generated the first transgenic ‘Astaxanthin Rice’ capable of synthesizing astaxanthin (Zhu et al. 2018). For comparative analysis, the investigators also constructed vectors encompassing the genes *sZmPSY* and *sPaCrtI*, as well as four genes encoding sZmPSY1, sPaCrtI, sCrBKT and sHpBHY, respectively, resulting in the production of β-carotene-enriched Golden Rice and canthaxanthin-enriched Rice.

### Biosynthesis of betalaines

**Fig. 4:**
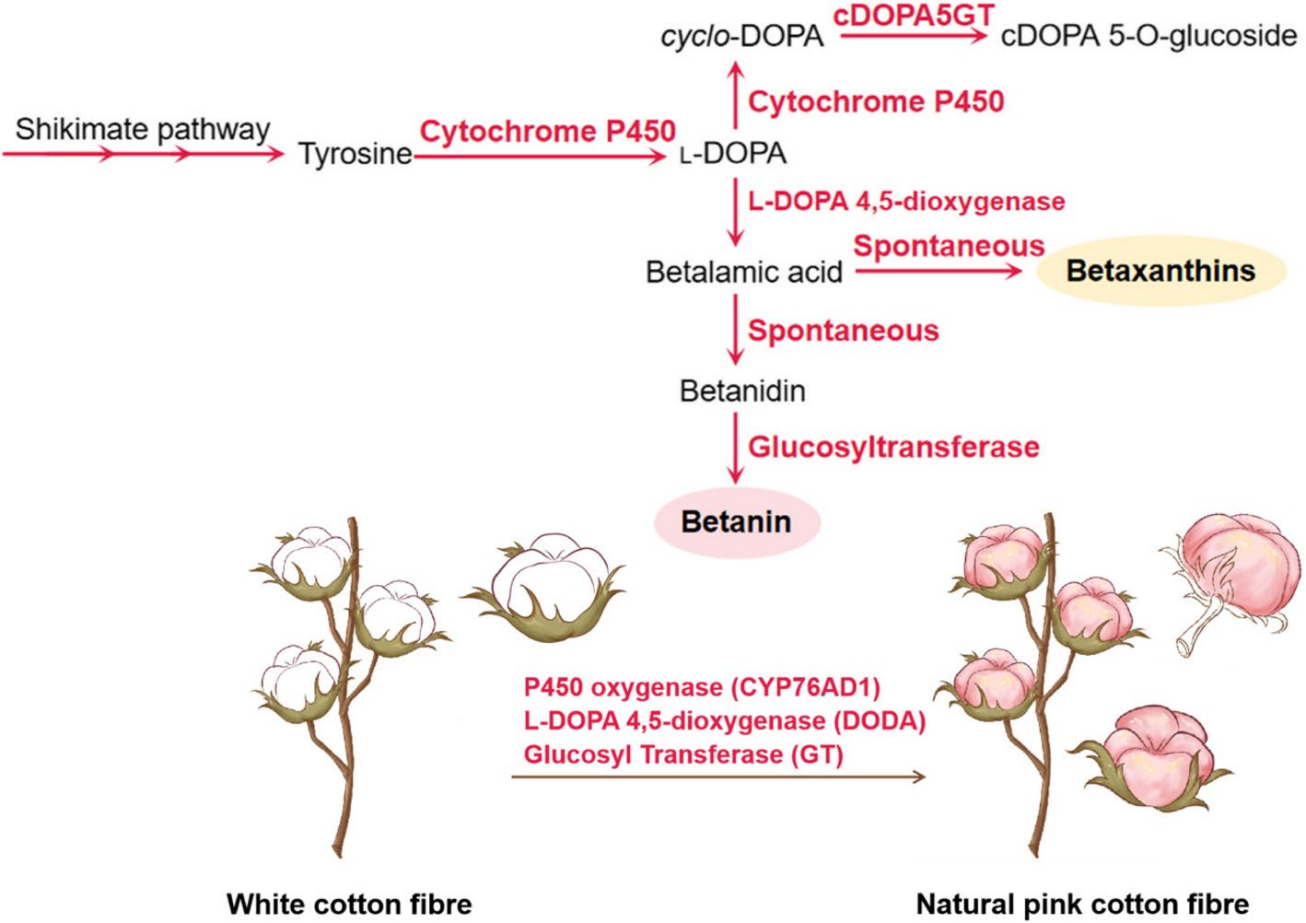
Betalaines biosynthetic pathways

Unlike flavonoids and carotenoids, which are ubiquitous in most plants, betalaines only exist in some core species of *Caryophyllales*. Therefore, the biosynthetic pathways of betalains and the enzymes and genes involved in the pathway are much less reported than those of flavonoids and carotenoids. The biosynthesis process of betalains, predicated to use tyrosine as the substrate, encompasses a multitude of steps involving both enzymatically catalyzed reactions and spontaneous chemical transformations (Gandía-Herrero and García-Carmona 2013) (Fig. 4). To address the environmental pollution and health concerns caused by the extensive use of chemical dyes in cotton textile processing, genetic engineering offers a promising solution by creating various colored cotton fiber types. However, the current-colored cotton varieties, mainly brown and green, have limited color diversity and fail to meet the diverse and vibrant demands of consumers. Recently, Ge et al. (2022) applied a remarkable approach to fabricate naturally pink-hued fiber using several key genes, *CYP76AD1*, *DODA*, and *GT,* coming from *Beta vulgaris*, necessary for betalain biosynthesis. Sublime achievements were made without jeopardizing the yield or quality of the cotton, thus offering an ecologically sustainable and health-beneficial substitute to artificial dyes for the production of chromatic cotton fibers. The discovery pioneers an innovative pathway in sustainable manufacturing, emphasizing the profound implications of plant biotechnology in the textile industry.

### Biosynthesis of chlorophylls

**Fig. 5:**
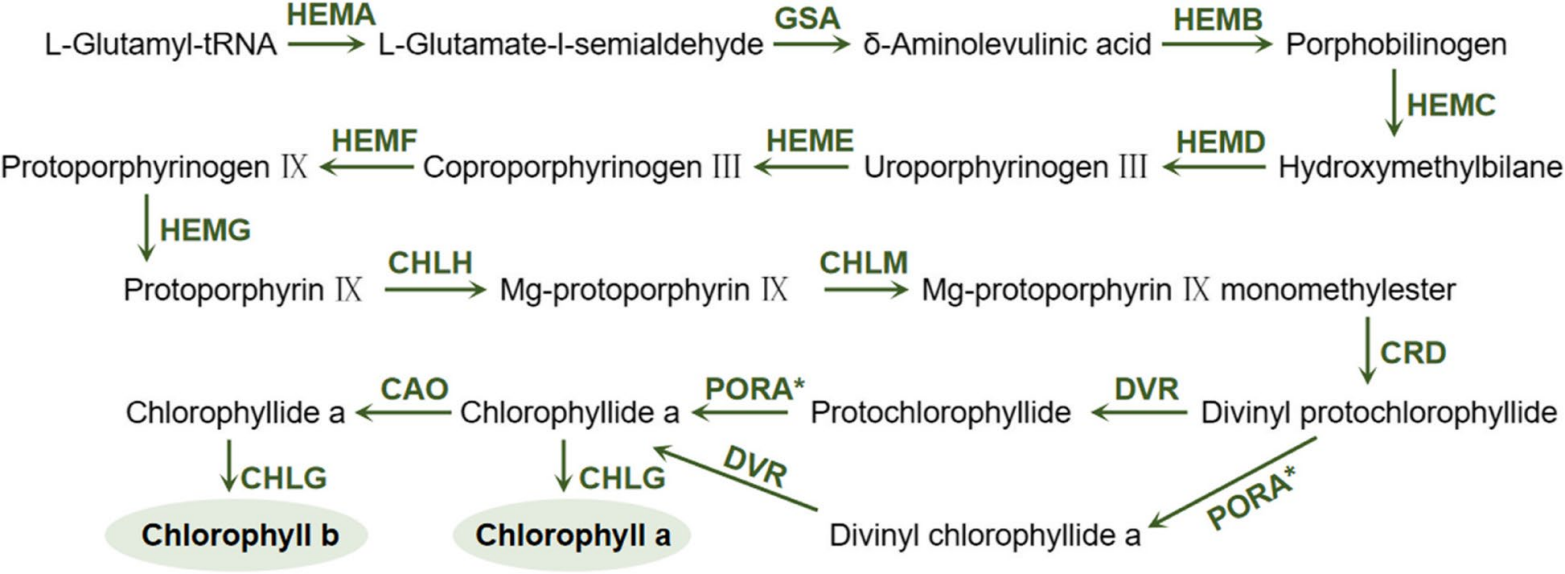
Chlorophylls biosynthetic pathways (Beale 2005). HEMA, Glutamy tRNA reductase; GSA, Glutamate1-semialdehyde minotransferse; HEMB, 5-Aminolevulinate dehydratase; HEMC, Hydroxymethylbilane synthase; HEMD, UroporphyrinogenIII synthase; HEME, UroporphyrinogenIII decarboxylase; HEMF, Coproporphyrinogen oxidative decarboxylase; HEMG, Protoporphyrinogen oxidase; CHLH, Mg-chelatase(I1, I2, D, H subunit); CHLM, Mg-protoporphyrinIX methyltransferase; PORA, PORB and PORC, NADPH protochlorophyllide oxidoreductase; DVR, Divinyl reductase; CHLG, Chlorophyll synthase; CAO, Chlorophyllide a oxygenase

A variety of chlorophyll types exist, including chlorophylls a, b, c, and d, as well as bacterial chlorophyll, among others. In higher plants, chlorophyll a and b are the primary forms of chlorophyll involved in food-related processes. The structural hallmark common to all chlorophyll types is the presence of porphyrin rings consisting of four pyrrole units, each bound to a central magnesium atom. Over the past decade, significant progress has been made in identifying the genes responsible for encoding nearly all the enzymes involved in plant chlorophyll biosynthesis, and comprehensive characterization of their enzymatic activities has been achieved (Shen et al. 2022a). Figure 5 illustrates the biosynthetic pathway of chlorophyll is completed by a series of enzymatic reactions (Beale 2005). First, the δ-aminolevulinic acid (ALA) is utilized as a precursor for the biosynthesis of protoporphyrin IX (Proto IX). The author mentioned that Proto IX is a common precursor to form chlorophyll, heme, and their derivatives. It combines iron to form heme and magnesium to generate magnesium protoporphyrin. The magnesium protoporphyrin receives another methyl group, and after cyclization, it becomes protochlorophyll a (PChlide a), which is also called protochlorophyll. The latter forms chlorophyll a through photoreduction, esterification and other steps. In addition to chlorophyll a, chlorophyllide a can serve as a substrate for the biosynthesis of chlorophyll b, chlorophyll d, and chlorophyll f.

## Metabolic engineering

**Fig. 6:**
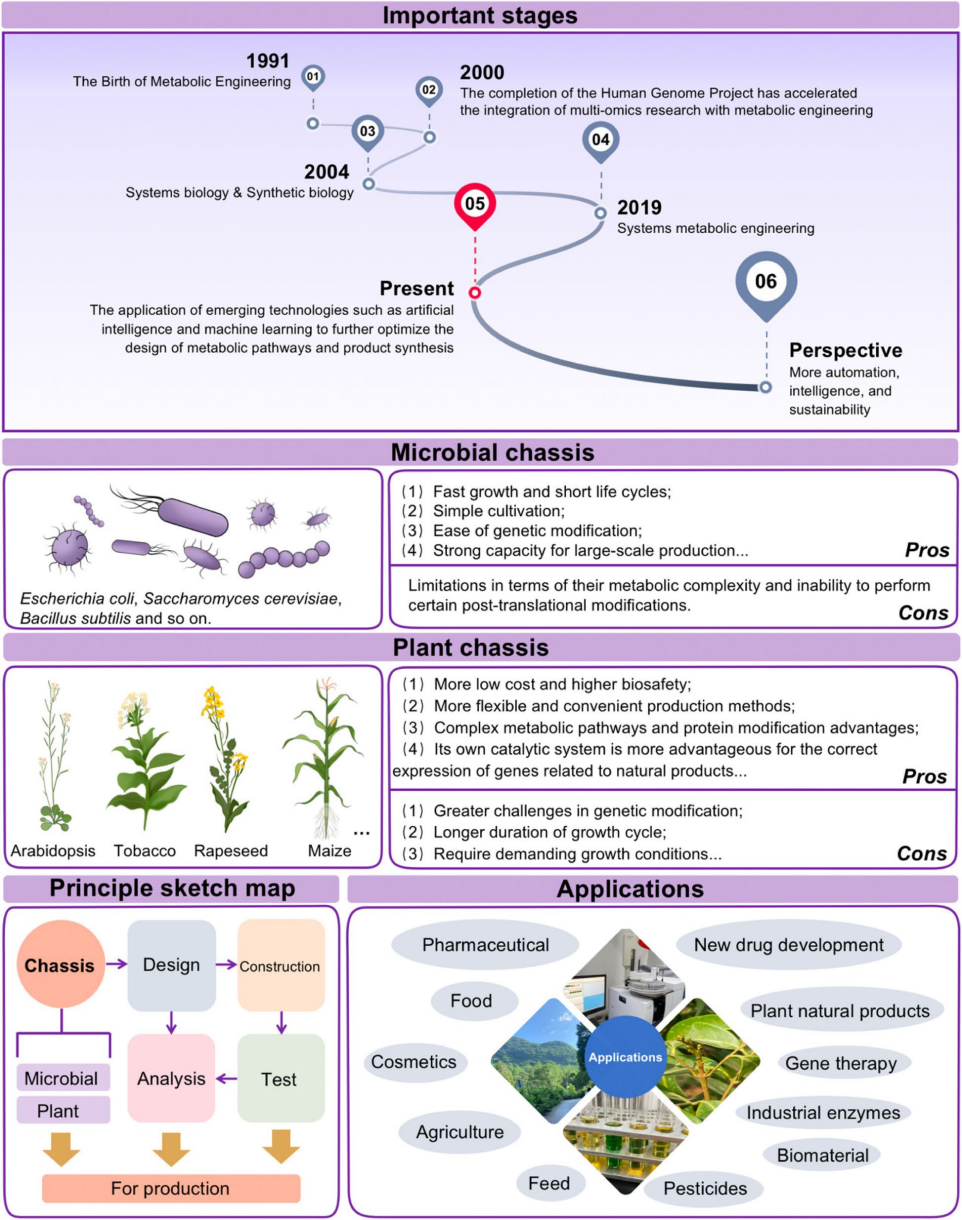
Important stages, main vectors and applications in metabolic engineering research. Created with Adobe Illustrator

Metabolic engineering uses recombinant DNA technology, gene and genome editing, and other technologies to modify the metabolic pathway of microbes or plants and other organisms in a targeted way to efficiently synthesize target products (Kim et al. 2023; Zhao et al. 2023). Host cells (also known as chassis cells), including microbial cells, plant cells, and animal cells, are important biological systems for metabolic engineering-related research (Fig. 6). Especially for biological manufacturing, different types of host cells are suitable to produce different molecules.

At present, microbial cells such as *Escherichia coli* and *Saccharomyces cerevisiae* are the main host cells. They are used widely in metabolic engineering because of their simple metabolite profiles and remarkable yield efficiency. For example, various studies supported engineering *Escherichia coli* for the production of pigments, such as (2S)-hesperetin, licoflavanone and chlorophyll, which are widely applied in food, chemical and pharmaceutical industries (Chen et al. 2018; Liu et al. 2023a; Zhang et al. 2024a). Meanwhile, previous studies also reported that the metabolic engineering for efficient anthocyanins, flavonoid xanthohumol and violaxanthin production in yeast (Mei et al. 2024; Wang et al. 2024; Yang et al. 2024a). Apart from the above, with the development of gene editing technology such as CRISPR/CAS system (Ma et al. 2023; Ren et al. 2023), more and more microbial cells such as *Corynebacterium glutamicum*, *Bacillus subtilis*, filamentous fungi, and non-traditional yeast have been widely used, effectively expanding the scope of host for metabolic engineering-related research (Tang et al. 2024). For example, earlier work successfully engineered the anthocyanin biosynthesis pathways using both CRISPR/Cas9 and CRISPRa systems in pear (Ma et al. 2023; Ming et al. 2022). Other studies reported that *Corynebacterium glutamicum* can be utilized as a platform to produce carotenoids and amino acids (Henke et al. 2018; Sumi et al. 2019). Furthermore, the highest lycopene content (21.08 μg/g FW) in tomato fruits with *Bacillus subtilis* was observed, indicating that *Bacillus subtilis* is a versatile bacterium beneficial to enhance antioxidant activity (Chandrasekaran et al. 2019).

Metabolic engineering based on plants is also in a phase of vigorous development. Compared with microbes, plants have natural advantages such as high safety, good enzyme adaptability and potential for industrial production. Tobacco is widely used as a plant chassis due to its rich secondary metabolites and good tolerance and transport ability to some metabolites. A study unveiled that the expression of key enzymes in the flavonoid pathway was significantly increased by co-overexpressing *AtMYB12* and *GmIFS1* in tobacco, leading to the synthesis of approximately 0.05 mg/g of genistein in the fresh tissues (Pandey et al. 2014). Furthermore, Li et al. (2019a) demonstrated that their engineered plants produced taxadiene and taxadiene-5α-ol, both of which are committed taxol intermediates, at the concentration of 56.6 μg/g FW and 1.3 μg/g FW, respectively. The recent availability of the full gene repertoire involved in the biosynthesis of the heptasaccharide triterpene glycoside bridgehead QS saponins and QS-7 has opened new avenues for future endeavors, aiming to enhance production in heterologous expression systems towards achieving commercially viable yields (Reed et al. 2023). These studies underpin the untapped potential of Nicotiana species as alternative platforms for metabolite production. Beyond *N. benthamiana*, there are other viable plant chassis that have been reported for metabolite production, including tomato (Li et al. 2022), potato (Liu et al. 2017), eggplant (Mishiba et al. 2020), petunia (Oliva et al. 2015), corn (Alameldin et al. 2017), and the increasingly promising poplar (Mottiar et al. 2023) (Fig. 6).

More and more successful cases of engineered fruits with vibrant colors and enhanced health benefits have been reported. For instance, the purple tomato, generated by expressing two transcription factors from snapdragon in tomato, was recently approved by the Food and Drug Administration (FDA) in the USA. The purple tomato accumulates more anthocyanins, which not only enhance its nutritional value but also exhibit potent antioxidant activity. Furthermore, it is confirmed that introducing four endogenous synthetic genes, *SlLCYE*, *SlLCYB*, *SlHYDB*, and *SlHYDE* under fruit-specific promoters can achieve lutein/zeaxanthin biosynthesis in tomato fruit (Wu et al. 2022b). In apple fruit, it is identified that using the apple transcription factor MYB10 can raise the polyphenolic content by metabolic engineering of the anthocyanin pathway (Espley et al. 2013). In strawberry fruit, allelic variation of MYB10 is the major factor controlling natural variation in flesh color (Castillejo et al. 2020).

In recent years, with the rapid development of frontier fields, including synthetic biology, genome editing, deep machine learning and automation technology, the field of metabolic engineering has been further pushed to an unprecedented level. A study provides a systematic overview of the remarkable contributions made by machine learning approaches (Kim et al. 2020). Within the domain of systems metabolic engineering, it is crucial to recognize and appreciate the indispensable role played by machine learning methodologies, as they have proven to be highly effective at every pivotal stage. The advent of machine learning has indubitably revolutionized the field, permeating and augmenting the efforts spanning across every pivotal facet of this discipline. From elucidating metabolic networks to optimizing metabolic pathways and from predicting metabolic behaviors to designing novel enzyme functions, machine learning techniques have unraveled unprecedented opportunities for exploring and manipulating cellular metabolism. Additionally, several small-scale microorganism fermentation techniques, in conjunction with automation, have enhanced the throughput of enzyme and strain phenotyping experiments (Raj et al. 2021). In fact, plant metabolic engineering has made significant progress in various aspects, including enhancing plant disease resistance, improving yield and quality, synthesizing pharmaceuticals and chemicals, and enhancing nutritional value. These achievements have made important contributions to food security, drug production and sustainable development. However, exploring the impact of pigment composition and concentration on the bioactivity of derivative metabolites remains a significant challenge that promises substantial medical implications. With regard to methodological tools, especially gene editing and enzymatic engineering, there clearly exists a persistent demand for their enhanced precision and evolution (Zhao et al. 2022).

## Conclusions and future prospects

Colorful fruits evoke a range of sensory experiences, from passionate reds to warm oranges, fresh greens to pure whites, and mysterious blue-blacks. The color of fruits depends on the variety and content of pigments, which are natural bioactive substances with nutritional value and potential pharmacological effects. Notably, phytochemicals have been the subject of numerous studies exploring approaches to modify pigment metabolism for nutritional or industrial enhancement of fruits through metabolic engineering. However, a comprehensive understanding of the mechanisms underlying these effects remains elusive. In this review, we systematically summarize the main pigment types contained in five kinds of fleshy fruits with different colors, functions, applications, biosynthetic pathways, and metabolic engineering, providing a reference for the biosynthesis of natural pigments and the terms of industrial and medical applications.

Fruits often contain multiple types of pigments simultaneously, and each pigment type can consist of diverse chemical compounds with varying structures. The type and quantity of pigments in fruits contribute to their distinct colors. It is noteworthy that white fruits are often devoid of pigments or contain only a trace amount of pigments, rendering them imperceptible to the human eye. Numerous in vitro and in vivo studies have demonstrated the health-promoting efficacy of natural pigments. For instance, the ingestion of red fruits has been associated with cardiovascular and cerebrovascular protection, while orange/yellow fruits are purported to benefit eye and skin health. Green fruits have been linked to liver health and nerve protection, and white fruits have been linked to bone and cardiovascular health. Additionally, blue-black fruits have been suggested to contribute to eyesight preservation and prevention of senile dementia. Despite the increasing body of knowledge on the diverse functions of pigments, further clinical experimental studies are warranted to establish the optimal dosages, efficacy, and bioavailability of these compounds. Moreover, the utilization of metabolic engineering techniques for the generation of novel and health-promoting pigment compounds requires further research.

Due to the presence of different types of pigments, fruits of the same species, such as pitaya, strawberry, and mulberry, can exhibit varying flesh colors. In the case of pitaya, the red-fleshed variety contains betacyanin pigments, which contribute to its distinctive color, while the white-fleshed variety lacks these pigments. In the market, red-fleshed pitaya often commands a higher price compared to white-fleshed pitaya. This price difference can be attributed to several factors. Firstly, red-fleshed pitaya is believed to contain a richer profile of nutrients, including antioxidants, vitamins, and dietary fiber, which contribute to its perceived higher quality and nutritional value, justifying the premium price. Secondly, the vibrant color of red-fleshed pitaya may be associated with superior quality and visual appeal, thus enhancing its market positioning and consumer desirability. Additionally, the limited availability of red-fleshed pitaya in the market, possibly due to lower production or inadequate supply, can further drive up its price. Lastly, consumer preferences for taste, flavor, or visual appearance may also influence their willingness to pay a higher price for red-fleshed pitaya. Nevertheless, to fully understand the impact of fruit color on price and market positioning, further rigorous research is warranted.

## Data Availability

Not applicable.

## Abbreviations

4CL: 4-Coumarate CoA ligase
C4H: Cinnamic acid 4-hydroxylase
CAO: Chlorophyllide a oxygenase
CHLG: Chlorophyll synthase
CHI: Chalcone isomerase
CHS: Chalcone synthase
CHYB: β-Carotene hydroxylase
CRTISO: Carotenoid isomerase
CYP: Cytochrome P450 enzymes
DMAPP: Dimethylallyl diphosphate
DVR: Divinyl reductase
F3H: Flavanone 3-hydroxylase
F3′H: Flavanoid 3′-hydroxylase
F3′5′H: Flavanone 3′,5′-hydroxylase
FLS: Flavonol synthase
FNS: Flavone synthase
GGPP: Geranylgeranyl diphosphate
GGPPS: GGPP synthase
GSA: Glutamate1-semialdehyde minotransferse
HEMA: Glutamy tRNA reductase
HEMB: 5-Aminolevulinate dehydratase
HEMC: Hydroxymethylbilane synthase
HEMD: UroporphyrinogenIII synthase
HEME: UroporphyrinogenIII decarboxylase
HEMF: Coproporphyrinogen oxidative decarboxylase
HEMG: Protoporphyrinogen oxidase
HID: 2-Hydroxyisoflavanone dehydratase
IFS: Isoflavone synthase
IPI: Isopentenyl diphosphate isomerase
IPP: Isopentenyl diphosphate
LAR: Leucoanthocyanidin reductase
LCYB: Lycopene β-cyclase
LCYE: Lycopene ε-cyclase
LDOX: Leucoanthocyanidin dioxygenase
NXS: Neoxanthin synthase
PAL: Phenylalanine ammonia-lyase
PDS: Phytoene desaturase
PSY: Phytoene synthase
UFGT: UDP-glucose flavonoid 3-O-glucosyltransferase
ZDS: ζ-Carotene desaturase
ZEP: Zeaxanthin epoxidase
Z-ISO: ζ-Carotene isomerase
